# Discriminating and classifying odontocete echolocation clicks in the Hawaiian Islands using machine learning methods

**DOI:** 10.1371/journal.pone.0266424

**Published:** 2022-04-12

**Authors:** Morgan A. Ziegenhorn, Kaitlin E. Frasier, John A. Hildebrand, Erin M. Oleson, Robin W. Baird, Sean M. Wiggins, Simone Baumann-Pickering

**Affiliations:** 1 Scripps Institution of Oceanography, University of California San Diego, La Jolla, California, United States of America; 2 NOAA Fisheries Pacific Islands Fisheries Science Center, Honolulu, Hawaii, United States of America; 3 Cascadia Research Collective, Olympia, Washington, United States of America; Wildlife Conservation Society Canada, CANADA

## Abstract

Passive acoustic monitoring (PAM) has proven a powerful tool for the study of marine mammals, allowing for documentation of biologically relevant factors such as movement patterns or animal behaviors while remaining largely non-invasive and cost effective. From 2008–2019, a set of PAM recordings covering the frequency band of most toothed whale (odontocete) echolocation clicks were collected at sites off the islands of Hawaiʻi, Kauaʻi, and Pearl and Hermes Reef. However, due to the size of this dataset and the complexity of species-level acoustic classification, multi-year, multi-species analyses had not yet been completed. This study shows how a machine learning toolkit can effectively mitigate this problem by detecting and classifying echolocation clicks using a combination of unsupervised clustering methods and human-mediated analyses. Using these methods, it was possible to distill ten unique echolocation click ‘types’ attributable to regional odontocetes at the genus or species level. In one case, auxiliary sightings and recordings were used to attribute a new click type to the rough-toothed dolphin, *Steno bredanensis*. Types defined by clustering were then used as input classes in a neural-network based classifier, which was trained, tested, and evaluated on 5-minute binned data segments. Network precision was variable, with lower precision occurring most notably for false killer whales, *Pseudorca crassidens*, across all sites (35–76%). However, accuracy and recall were high (>96% and >75%, respectively) in all cases except for one type of short-finned pilot whale, *Globicephala macrorhynchus*, call class at Kauaʻi and Pearl and Hermes Reef (recall >66%). These results emphasize the utility of machine learning in analysis of large PAM datasets. The classifier and timeseries developed here will facilitate further analyses of spatiotemporal patterns of included toothed whales. Broader application of these methods may improve the efficiency of global multi-species PAM data processing for echolocation clicks, which is needed as these datasets continue to grow.

## Introduction

The Hawaiian archipelago creates a regional oasis in the oligotrophic waters of the North Pacific Subtropical Gyre [1–3]. The area is an attractive habitat for large ocean predators including odontocetes, or toothed whales. At least 18 species of odontocetes reside in the region [4], several of which have island-associated stocks [5]. These stocks in particular have limited geographic ranges and may be especially vulnerable to environmental perturbations and anthropogenic impacts (e.g. [5]).

Passive acoustic monitoring (PAM) in odontocete study and conservation efforts can provide long-term, non-invasive, and cost-effective continuous monitoring of these species (e.g., [6–8]). Scientists employ a variety of acoustic recording schemes for PAM monitoring, including various bottom-moored hydrophones systems [6, 9] and shipboard studies using towed acoustic arrays with combined visual observations [10, 11]. Bottom-moored equipment has the advantage of continuous recording over long time periods, but can suffer from difficulties in distinguishing species, resulting in few multi-species analyses. Towed array studies can cover a spatially diverse range, often with species verification, but are usually temporally limited due to factors including weather and the cost of ship time. These efforts can include analyses of a variety of animal signals including tonal vocalizations (e.g., whistles [6, 12]) and echolocation clicks, which are produced by odontocetes for foraging and navigational purposes [13, 14].

Echolocation clicks are particularly useful in PAM as certain click types are produced exclusively by a single species and under a wide variety of behavioral states, resulting in a useable proxy for animal presence. PAM data can be utilized for reliable detection of species for which species-specific echolocation click types have been identified (e.g., [15, 16]). For this purpose, feature vectors of echolocation clicks, typically including the timing between successive clicks in an echolocation click ‘train’ (inter-click-interval, or ICI), spectral properties including peak frequency and spectral shape, and properties of the waveform (e.g., number of oscillations, waveform envelope and overall duration), are used to discriminate between odontocete species using automated algorithms [17].

Long-term PAM data has been used in recent years to determine distributions and densities of odontocetes as well as provide information on behaviors from diving and diel foraging patterns to larger patterns of animal movement [6, 7, 18–20]. PAM data has also been shown to be valuable and well-suited for habitat modeling of a variety of cetacean species [21–23]. The NOAA Pacific Islands Fisheries Science Center (PIFSC) has been collecting passive acoustic monitoring data with recordings covering the dominant frequency band of most odontocete echolocation clicks (i.e., 5–100 kHz) for the past decade at three Hawaiian sites. While some studies have utilized portions of this dataset (e.g., [24, 25]), the full dataset has not been analyzed to identify the full suite of species present.

The ability to detect and classify echolocation clicks within such a large dataset has been limited by the time-intensive nature of the manual classification approaches previously required to derive time series of acoustic presence for a given species. In recent years, machine learning tools have been successfully used in detection and discrimination tasks for a variety of species and ocean basins (e.g., [6, 26, 27]). Deep neural networks (e.g., [28, 29]), random forests (e.g., [30, 31]), and clustering algorithms (e.g., [32, 33]), as well as a variety of other classification regimes, have all been used for these purposes. Amongst these techniques, unsupervised clustering (e.g., [13, 17]) in particular can expedite the processing of echolocation clicks in large PAM datasets by allowing for the automated distillation of dominant signal types. These methods make use of click features to cluster similar clicks and present an opportunity to identify both known and novel signals. These signals can then be attributed to species using literature records of echolocation clicks and auxiliary data such as sighting records, tag data locations, or towed acoustic array data with concurrent visual observations [34]. Attribution of echolocation clicks to species in this way facilitates development of large training sets for species identification. This method has been used successfully to analyze data from the Gulf of Mexico [17] for a variety of species but has not yet been applied to the Hawaiian Islands region. Once derived, these types can be used as input classes to build a neural-network based classifier that can be run on novel data and hence expedite analyses of large acoustic datasets [35].

Some species of odontocetes present in the Hawaiian Islands region, such as Cuvier’s beaked whale, *Ziphius cavirostris*, and Blainville’s beaked whale, *Mesoplodon densirostris*, produce echolocation clicks which have already been described in the literature [16], while others remain acoustically uncharacterized. Even when clicks have been described, limitations of available classification methods in correctly identifying the whole suite of species’ clicks hinders processing of datasets for the full repertoire of local species at once, especially when signals are highly similar. For some species or species groups, such as beaked whales and *Kogia* spp., disparate detectors have been used to successfully identify and study target species (e.g., [36, 37]). In this study, machine learning methods were used for signal discovery, detection, and classification of Hawaiian Islands regional PAM data, resulting in a comprehensive library of the dominant echolocation click types present at three monitoring sites. A neural network-based classifier was then developed and used to classify clicks across the entirety of this dataset, facilitating future regional studies of included species. The tools used here improve upon previous methodologies by allowing for a single detection step and classification workflow for all included species, expediting data processing. Additionally, the methodology employed is malleable in that the classifier learns from the data itself instead of using heuristics defined by other researchers, often in differing ocean basins, to define types.

## Methods

### Data collection

Passive acoustic data were collected using bottom-moored High-frequency Acoustic Recording Packages (HARPs) [38] consisting of one or more hydrophones, logging equipment, batteries, and flotation. The majority of the deployments used in this study utilized a system consisting of a low-frequency and a high-frequency hydrophone, with the crossover between the two occurring at either 2 or 25 kHz depending on the deployment (S1 Table). Crossover frequency is important to note as changes in sensitivity at the crossover frequency can affect analyses. In all cases, sensors were connected to custom-built preamplifiers and bandpass filters. Frequency-dependent sensitivity of representative systems was calibrated at the Navy’s Transducer Evaluation Center (TRANSDEC). Locations of specific deployments varied slightly due to the difficulty of at-sea deployment of seafloor moorings.

**Fig 1:**
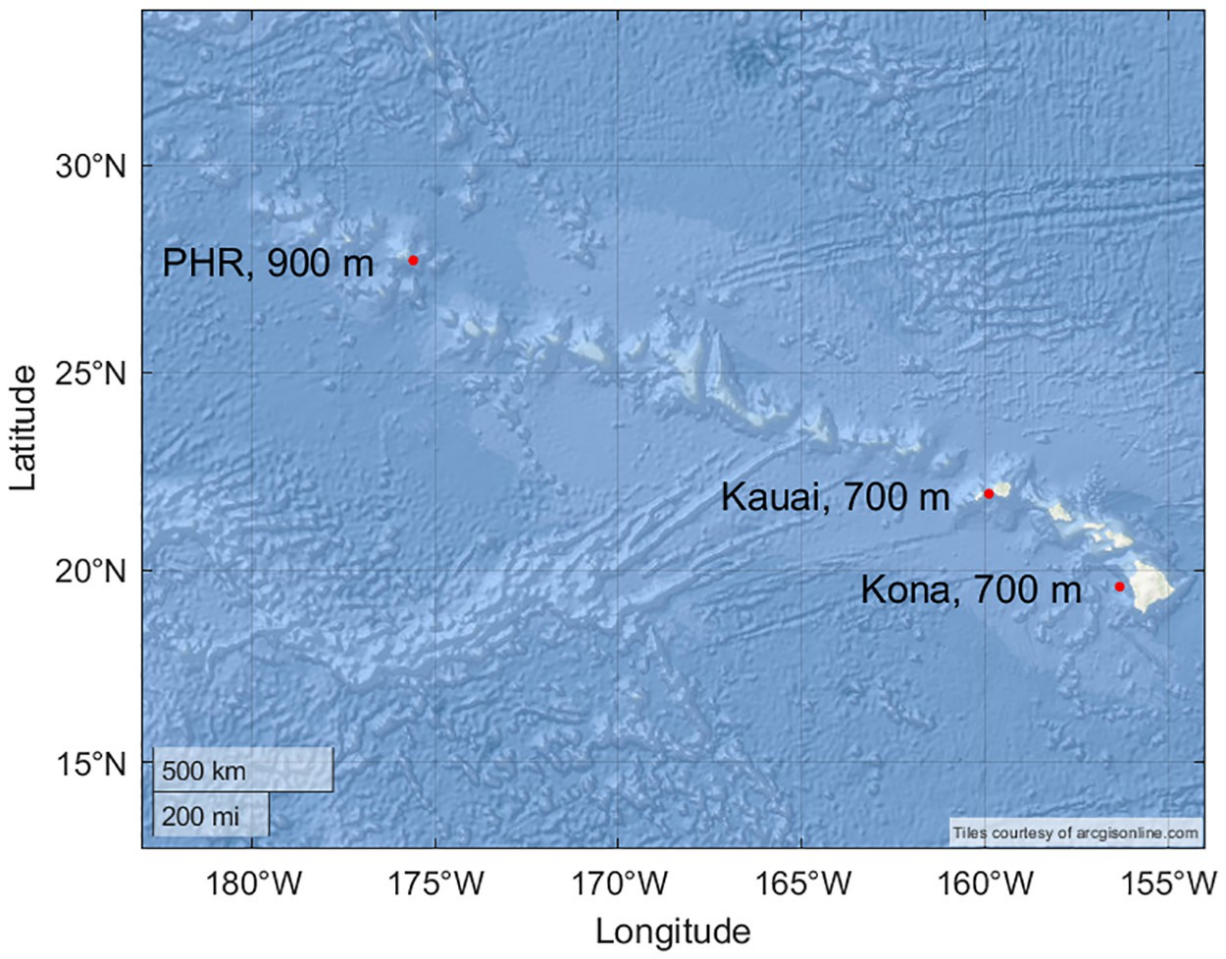
Map of recording locations. Map showing the latitude-longitude locations of the Kona, Kauaʻi, and PHR sites. Location and depth of each site was averaged among deployments from that site. Basemap image is the intellectual property of Esri and is used herein with permission. Copyright © 2022 Esri and its licensors. All rights reserved.

Data from three recording sites were included in this study: one off the west side of Hawaiʻi Island (henceforth referred to as Kona), one off the western side of Kauaʻi, and one on the northern side of Manawai (also known as Pearl and Hermes Atoll and henceforth referred to as PHR) (Fig 1; S1 Table). Deployment setup varied at these sites in terms of recording schedule, instrument depth, and duty cycle regime (S1 Table).

Duty cycling refers to alternating periods of recording and non-recording (e.g., recording of 5 minutes out of every 25 minute period) to extend battery life and allow for longer deployments. Data from these sites were recorded at a 200 kHz or 320 kHz sampling frequency and 16 bit quantization at depths ranging from 550–1150 meters (S1 Table). All hydrophones were buoyed approximately 10–30 meters from the seafloor. The data from the three sites combined represents approximately 15 instrument years of recordings (S1 Table).

### Data processing

#### Click detection

Odontocete echolocation clicks were detected using an energy detector that ran as an added package of the MATLAB-based software program *Triton* [39]. This detector first determined periods for which acoustic energy in a frequency band of interest exceeded a user-defined threshold, then searched those time periods for impulsive signals that met several criteria characterizing odontocete echolocation clicks. The underlying mechanics of the detector are described in Frasier et al. (2017) [17] and in Frasier, K.E. (2021) [35] in more detail. Specifications for this detector included application of a high pass filter at 10 kHz to exclude low frequency noise sources. A low pass filter was set at 100 kHz, regardless of sampling frequency, to simplify clustering and neural network steps. Additionally, a peak-to-peak amplitude threshold of 115 dB_pp_ re 1 μPa was set after manual review of a subset of the data determined that this was an acceptable threshold to consistently detect a majority of odontocete clicks while excluding most low amplitude impulsive signals from ships and other noise sources. Signal duration was used to exclude non-target signals; only detections between 30–1200 μs in duration were retained, and detections with less than 100 μs of separation were merged. Features of retained clicks including time and date, peak-to-peak received level, and frequency spectra were stored for subsequent analyses. Frequency spectra were calculated using a 400-point (sampling frequency (fs) = 200 kHz) or 640-point (fs = 320 kHz) FFT of Hanning-windowed data centered on the click peak amplitude.

#### Unsupervised clustering methods and click type identification

Detected impulsive signals were separated into distinct types via a two-step clustering method. At this point, a minimum received level of 120 dB_pp_ re 1 μPa was set to perform clustering on higher-quality detections. In the first clustering step, data were split into 5-minute bins. Individual detections in each bin that passed the received level threshold were compared against one another and clustered together based on spectral shape (pairwise correlation distance, [40]) using the Chinese Whispers (CW) algorithm [41]. This process was iterative, with each detection within a 5-minute bin beginning as a single-node cluster and being iteratively re-assigned to larger, closely related clusters until reassignment ceased (i.e., until all detections were assigned the same label as detections to which they were most strongly connected). Minimum cluster size was set at 50 detections, and maximum network size was set at 10,000 nodes (chosen at random from all detections in the bin) due to computational limitations. A maximum of 15 iterations of this process were completed, though clustering usually ceased before reaching this threshold. The final partition for each bin was chosen based on highest average normalized mutual information (NMI) score [42], which compares clusters across multiple partitions to determine consistency of types.

Though not used as a feature for clustering, ICI distributions were calculated for retained bins for use in later classification steps. These distributions were calculated for each cluster found in a 5-minute bin by calculating the timing between successive detections in that cluster, with distributions being truncated at 0.6 seconds. This value was inclusive of known modal inter-click interval values for target odontocete species. ICI distribution shape and modal ICI values were used in later evaluation steps instead of ‘raw’ ICI values between detections to overcome issues related to recording multiple animals instead of a single individual.

Mean normalized spectra and mean normalized waveform envelope were also calculated for each cluster in every bin. To calculate mean normalized spectra, power spectral density of clicks was computed and converted to a dB scale. These spectra were normalized by setting the minimum amplitude to zero and maximum to one. Normalized spectra were then averaged to determine mean normalized spectra on a dB scale (henceforth ‘mean spectra’).

In the second step, mean spectra and waveform envelopes determined in the first clustering step were compared across a large subset of the data to determine the dominant detection types in each deployment, again using pairwise distances and the CW algorithm. Thresholds for this step were similar to those used in the first clustering step, though in this case the maximum allowable network size was 20,000 bins, and retained clusters were required to contain a minimum of at least 25 5-minute bins. In this step, 1% of the least-connected nodes were pruned from within each cluster to result in cleaner final clusters. This second clustering step was performed for a total of five trials, with the best partition being chosen automatically based on NMI. More detail on this process can be found in Frasier, K.E. (2021) [35].

Once clusters were determined for each deployment, detected signals were visualized using *LabelVis*, a custom script developed by the author as an add-on package to *Triton* software [39] that allows users to visualize various depictions of the acoustic data overlaid with manually or automatically generated labels. This program is publicly available on GitHub [43] and allowed for manual examination of timeseries and spectral information for individual clicks from retained clusters to determine which might be combined or discarded. For each deployment, several months of data were examined to be sure that signals contributing to each cluster were distinct from others and not data artifacts. Clusters from a given deployment were determined to be the same if they were spectrally similar, had similar ICI distributions and modal ICI values, and were often concurrently or sequentially present. Some clusters (approximately 7% of total clusters) were alternatively determined to be mixed based on spectral or ICI similarities and high co-occurrence with multiple, distinct types. Such clusters were not grouped into the final types and did not contain any types that weren’t also found in non-mixed clusters. Clusters were then compared across deployments and sites and were grouped based on spectral similarity as well as shape of the ICI distribution to determine a final set of echolocation click types, as well as an outgroup of noise detections.

Final echolocation click types were described in terms of frequency and -3 dB bandwidth of their spectral peaks as well as peak ICI values using a subset of clicks from all three sites determined by inspection to be representative of the overall variability in spectra and ICI. Peaks and -3dB bandwidths were found using the main peak of individual click spectra for all available high-quality clicks of a given type (minimum = 2500 clicks). Click quality was determined by manual review, with a focus on removing detections for which spectra were subject to data artifacts. The distribution of these values was plotted and considered in conjunction with mean spectra for the type in order to determine whether or not multiple peaks should be described (i.e., whether the distribution of peak values was unimodal). ICI distributions were constructed and fit with Gaussian curves to determine the peak ICI value and approximate standard deviation. Where possible based on previous literature, click types were attributed to specific odontocetes at the genus or species level as detailed below.

#### Classifier creation and evaluation

For the purposes of classifier training and testing, the echolocation click types distilled above were used as input classes. An additional ‘junk’ class containing clusters of detections from noise sources such as ships and echosounders was included to prevent misclassifications of these sources as odontocetes. These noise sources were picked up by the detector due to their commonality in the data, particularly at the Kona site [44], and the similarity of these signals to echolocation clicks. Clusters of sperm whale, *Physeter macrocephalus*, echolocation clicks were also grouped into this ‘junk’ class due to the difficulty of separating these clicks from high-frequency ship noise as both can occupy the same frequency range between ~5–20 kHz. Additionally, there was a high likelihood of missing many sperm whale clicks beneath the lower end of the 10 kHz bandpass filter employed in click detection.

For each class, 5000 examples (e.g., 5000 bins from each final click type) were chosen at random from the input set and used in the train/test/validation data with a 70/20/10 split. For the class representing Cuvier’s beaked whale, input vectors of mean spectra, waveform envelope, and ICI values were augmented with additional example bins from similar data recorded off the coast of Southern California due to a limited number of observations in the Hawaiʻi dataset. Approximately 50% of the final train/test/validation examples for Cuvier’s beaked whale were from this additional dataset. Training, testing, and eventual labelling of novel data was completed at the 5-minute bin level based on groupings made in the first step of the clustering algorithm, i.e., all clicks clustered as one ‘type’ at the 5-minute bin level received the same label from the network. Labelling at this level allowed for the inclusion of ICI distribution as an input feature and tends to lead to higher classification accuracy [35]. Where the total number of example bins was less than 5000, existing examples were chosen at random and data were augmented to create “new” examples for the classifier to learn from. For mean spectra and waveform envelope, low-amplitude Gaussian white noise was generated and added to existing examples to create these “new” examples. For ICI, data points were augmented via addition of random values selected from a distribution generated from original input ICI values.

Click features used in network training were ICI distribution, spectral shape, and waveform envelope. A single classifier was developed using data from all sites. The network itself was compiled using an add-on package of *Triton* [39] that allows the user to construct a deep feed-forward neural network with user-specified parameters such as total number of epochs, size and number of hidden layers, and dropout rate. Networks with variations in the above parameters were compiled and evaluated based on accuracy for each type as well as confusion amongst types in both the training and testing sets. The top three networks were chosen from these based on minimizing confusion and maximizing accuracy across types. These networks were evaluated based on performance on novel data compared to manual labelling of that data. Novel data were chosen as a pseudo-random subset of all available data, and manual labelling of this dataset was completed using *DetEdit*, a graphical user interface for annotating acoustic events [45].

Performance was evaluated based on the accuracy, precision, misclassification rate, specificity (proportion of true negatives), and recall of each network to each class both at individual sites as well as the three sites combined. A final network was chosen based on highest accuracy, recall, and precision values across types and sites (Eqs 1 and 2). For the final chosen network, additional comparisons of network results versus manually labelled data were undertaken so that final performance metrics would cover the widest number of months and years possible at each site. Only bins containing clicks with a received level above 125 dB_pp_ re 1 μPa were considered in this final evaluation to account for differing sensitivities across hydrophones and sites. Performance on the novel data was again evaluated using accuracy, recall, and precision (Eqs 1–3). These metrics were evaluated by class and by site, as well as for all sites combined.


$$Accuracy=\frac{true\;positives+true\;negatives}{all\;detections}$$ (1)

$$Recall=\frac{true\;positives}{true\;positives+false\;negatives}$$ (2)

$$Precision=\frac{true\;positives}{true\;positives+false\;positives}$$ (3)

Resulting classifications on the full dataset were used to provide relative acoustic presence estimates of each class at each site, to bolster species or genus assignments via comparison with established sighting, tag, and acoustic records. All detections, including those from mixed clusters, received a label in this analysis step. Relative acoustic presence estimates were calculated per deployment as the percentage of recording days with presence of a given type. Final relative acoustic presence was then calculated by taking the average percent of odontocete presence attributable to a class across deployments at a site.

#### Auxiliary data sources and type classification

For echolocation click type distilled in the clustering process without clear assignments from previous literature, additional data sources were included in analyses in an attempt to assign these types to species. Towed array data available from the NOAA Hawaiian Islands Cetacean and Ecosystem Assessment Survey (HICEAS) 2017 cruise [46] around the Hawaiian Islands was used in this process. This dataset consisted of visual sightings of animals in conjunction with concurrent acoustic recordings, allowing bioacoustic signals to be reliably matched to species. Acoustic data from this cruise was collected using two 3-channel hydrophone arrays connected by 100 meters of cable, towed 300 meters behind the ship. The same detector used on the HARP data was used again on this acoustic data to examine clicks present during encounters where relevant species (i.e., rough-toothed dolphins, *Steno brednanesis*, common bottlenose dolphins (hereafter referred to as bottlenose dolphins), *Tursiops truncatus*, melon-headed whales, *Pepnocephala electra*, striped dolphins, *Stenella coeruleoalba*, pantropical spotted dolphins, *Stenella longirostris*, and spinner dolphins *Stenella attenuata*) were sighted. False positive detections during these encounters were removed using *DetEdit*. Concatenated and mean spectra were then calculated for these encounters for comparison to the potential click type.

Additional support for click type assignment was obtained from sighting data from the region of the Kona and Kauaʻi HARPs, obtained through boat-based sighting efforts [4]. When sightings occured near HARP locations, recorded clicks may be attributable to concurrently sighted animals. Sightings were assessed for any detections of relevant species that occurred within a 10 km radius of the HARPs. Sightings within this distance and within two hours of echolocation clicks labelled as the unknown click type were assessed for viability of providing a match based on the distance from the HARP and the time offset between the sighting and the acoustic encounter.

## Results

### Type classification

The click detection and clustering process resulted in ten echolocation click types, presumably representing ten or more species: false killer whale, *Pseudorca crassidens*, low-frequency 1 (LF1, possibly rough-toothed dolphin), short-finned pilot whale, *Globicephala macrorhynchus* (two click types), bottlenose dolphin/melon-headed whale, Blainville’s beaked whale, Cuvier’s beaked whale, stenellid dolphins (two click types), and dwarf (*Kogia sima*) or pygmy (*K*. *breviceps*) sperm whale (pooled as *Kogia* spp). Descriptive statistics for each type are provided in Table 1. Information on validation data used to attribute types to species is provided in Table 2. Available data for validation included previous acoustic records, spatial distributions (including abundance information), temporal behavior studies, and auxiliary sighting/acoustic data.

**Table 1:** Quantitative click type descriptions.

Neural Network Class, Number of Clicks	Spectral Peak 1 (kHz)		Spectral Peak 2 (kHz)		Spectral Peak 3 (kHz)		Modal ICI (milliseconds)
	Peak frequency	-3 dB bandwidth	Peak frequency	-3 dB bandwidth	Peak frequency	-3 dB bandwidth	
False killer whale, n = 4000	16.5 (13.0–20)	6.5 (1.0–12.0)	--	--	--	--	28.4 (+/- 28.0) 166 (+/- 109)
Low-frequency 1, n = 4000	22.0 (20–25)	5.5 (1.5–19.5)	--	--	--	--	169 (+/- 132)
Short-finned pilot whale 1, n = 7000	13.0 (12.0–13.5)	1.5 (1.0–6.5)	28.0 (26.0–31.0)	5 (2.0–10.0)	--	--	184 (+/- 66.9)
Short-finned pilot whale 2, n = 40000	13.0 (12.5–14.0)	1.5 (1.0–2.0)	18.5 (16.5–20.5)	3 (1.5–9.5)	48.5 (36.0–40.5)	3.0 (2.0–8.5)	206 (+/- 56.0)
Tt/Pe, n = 20000	12.5 (11.5–13.5)	1.5 (1.0–3.0)	32.5 (30.0–35.5)	5.5 (2.5–12.0)	--	--	109 (+/- 109)
Blainville’s beaked whale, n = 25000	24.0 (23.0–25.5)	2.5 (1.5–4.5)	36.0 (32.0–41.5)	9.0 (4.5–15.0)	--	--	319 (+/- 109)
Cuvier’s beaked whale, n = 2500	17.0 (16.0–18.5)	2.5 (2.0–3.5)	24.0 (22.0–25.5)	4.0 (2.0–9.0)	40.0 (37.0–44.0)	6.5 (3.0–12.5)	433 (+/- 59.0)
Stenellid 1, n = 100000	18.5 (16.5–20.5)	4.25 (3.0–9.75)	50.0 (45.0–54.0)	9.0 (3.5–18.5)	--	--	48.5 (+/- 43.5)
Stenellid 2, n = 90000	25.0 (22.5–27.0)	4.5 (3.0–6.5)	39.5 (35.0–44.5)	8.5 (4.0–18.0)	--	--	53.5 (+/- 0.0401)
*Kogia* spp., n = 6000	93.5 (87.5–99.5)	10.0 (5.0–17.5)	--	--	--	--	90.3 (+/- 41.8)

Parameters of click types (i.e. neural network classes) including median location of spectral peaks for all evaluated clicks as well as peak value for ICI distributions of evaluated acoustic encounters of each class. Species are organized based on overall frequency content of clicks (lowest to highest) and then by number of spectral peaks. Number of clicks evaluated is given along with class name. For spectral peaks, values in parentheses give the 10th and 90th percentiles of the data. For ICI, standard deviation from the peak (i.e. modal) value is given instead. For the false killer whale class, the ICI distribution was bimodal; in this case, two ICI values are given instead of one. The bottlenose dolphin/melon-headed whale type is abbreviated as Tt/Pe.

**Table 2:** Validation types.

Echolocation Click Type	Validation Type [References]
False killer whale	previous acoustic [26], spatial distribution [4, 27–29]
Low-frequency 1*	previous acoustic (limited) [33], spatial distribution [4, 34–35], temporal behavior [36], auxiliary sighting/acoustic data
Short-finned pilot whale 1	previous acoustic [16, 26], spatial distribution [4, 37, 38, 39]
Short-finned pilot whale 2	previous acoustic [16, 26], spatial distribution [4, 37, 38, 39]
Tt/Pe	previous acoustic [40], spatial distribution [4, 37, 43], temporal behavior [41–42]
Blainville’s beaked whale	previous acoustic [17], spatial distribution [4, 35, 44–46]
Cuvier’s beaked whale	previous acoustic [17], spatial distribution [3–4, 45]
Stenellid 1	previous acoustic [40, 47], spatial distribution [4, 28, 35, 37, 48, 49]
Stenellid 2	previous acoustic [40, 47], spatial distribution [4, 28, 35, 37, 48, 49]
*Kogia* spp.	previous acoustic [9, 50], spatial distribution [4, 51]

Validation sources (with references) for each echolocation click type. Validation types are previous acoustic, spatial distribution (including abundance information), temporal behavior, and auxiliary sighting/acoustic data. The Tt/Pe abbreviation corresponds to the bottlenose dolphin/melon-headed whale type.

* Validation of this type as likely rough-toothed dolphin is included in the main manuscript as this represented a novel type description. Further detail regarding all other types is available in S1 File.

#### A—False killer whale

**Fig 2:**
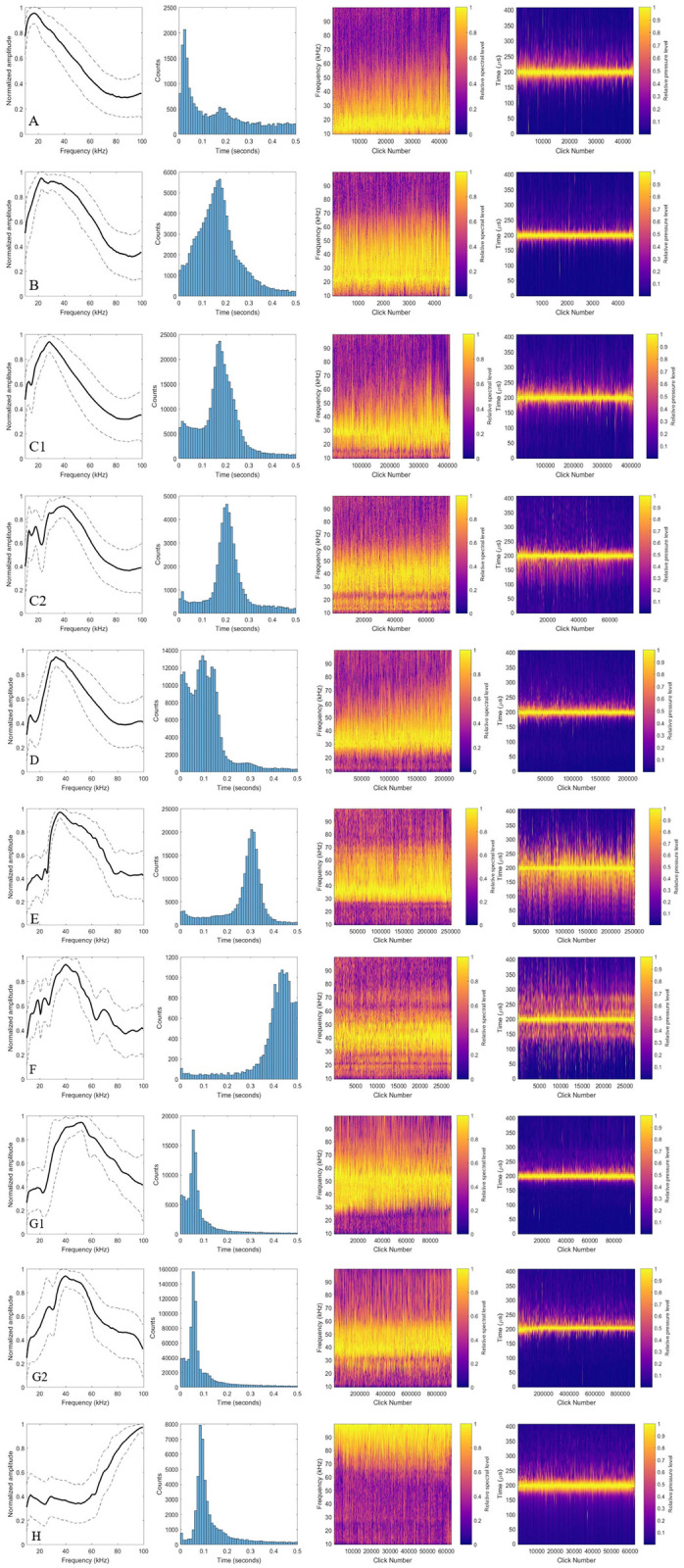
Echolocation click types. Plots A-H depicting data from representative clicks from each of 10 final click types: (A) False killer whale, (B) Low-frequency type 1 (LF1), (C1) Short-finned pilot whale 1, (C2) Short-finned pilot whale 2, (D) Bottlenose dolphin/ melon-headed whale, (E) Blainville’s beaked whale, (F) Cuvier’s beaked whale, (G1) Stenellid 1, (G2) Stenellid 2, and (H) *Kogia* spp. Panels 1–4 (left to right) depict the following: (1) mean spectra, shown along with 10^th^ and 90^th^ percentile values, (2) modal inter-click interval distribution, (3) concatenated click spectra of all clicks included, and (4) click waveform envelope for all clicks. Click waveform envelope has been sorted by peak amplitude (highest to the left), and concatenated clicks have been sorted correspondingly. Types are ordered by peak frequency.

The false killer whale echolocation click type was described by a single spectral peak at 16.5 kHz with -3 dB bandwidth of 6.5 kHz. The ICI distribution for this click type was bimodal, with a first peak at 28.4 ms and a second peak at 166 ms (Table 1, Fig 2A). The first peak in ICI was determined to be a result of multiple animals clicking at the same time, as well as single animals approaching a target. Labels of this type matched well with the encounters used for acoustic discrimination of false killer whales in Baumann-Pickering et al. (2015) [26]. For this type, acoustic presence determined from automated labelling was recalculated via manual checking of all false killer whale labels after manual review of labels revealed that many noise detections were being incorrectly labelled as this type. Final type assignment was based on previous acoustic and spatial records (Table 2, S1 File).

#### B—Low-frequency type 1 (LF1)—possible rough-toothed dolphin

The LF1 click type was described by a single spectral peak at 22.0 kHz with a -3 dB bandwidth of 5.5 kHz. The ICI distribution for this type had a single peak at 169 ms (Table 1, Fig 2B). This class did not match any well-established records of echolocation clicks, or have a match within click type ‘libraries’ previously produced using these clustering methods in other regions [17, 47–49]. However, the peak frequency and -3 dB bandwidth of this type were very similar to the limited previous descriptions of clicks of the rough-toothed dolphin [50]. This species has one of the highest abundance estimates of any in the Hawaiian Islands [51]. Additionally, small boat surveys over the course of 2000–2012 found >25% of all sightings of cetaceans near Kauaʻi, where LF1 is most common (present 83% of recording days), were attributable to rough-toothed dolphin [4]. This type was less common at the Kona and PHR sites (acoustically present 40% of days in both cases). This general trend (most common near Kauaʻi) is reflected in the sighting record of rough-toothed dolphins, in which this species represents only ~10% of sightings leeward of Hawaiʻi Island [4]. Less data is available in the vicinity of PHR, though the species has been sighted previously in the area [51]. Recently updated habitat-based density models for Hawaiian odontocetes suggest that the locations of the Kauaʻi and PHR HARPs are within predicted regions of highest density for the rough-toothed dolphin [52]. Preliminary exploration into diel trends in this type revealed an overwhelming decrease in acoustic activity during daylight hours, which fits with a recent study of rough-toothed dolphin diving behavior that has suggested the species is more active during dusk/night [53].

**Fig 3:**
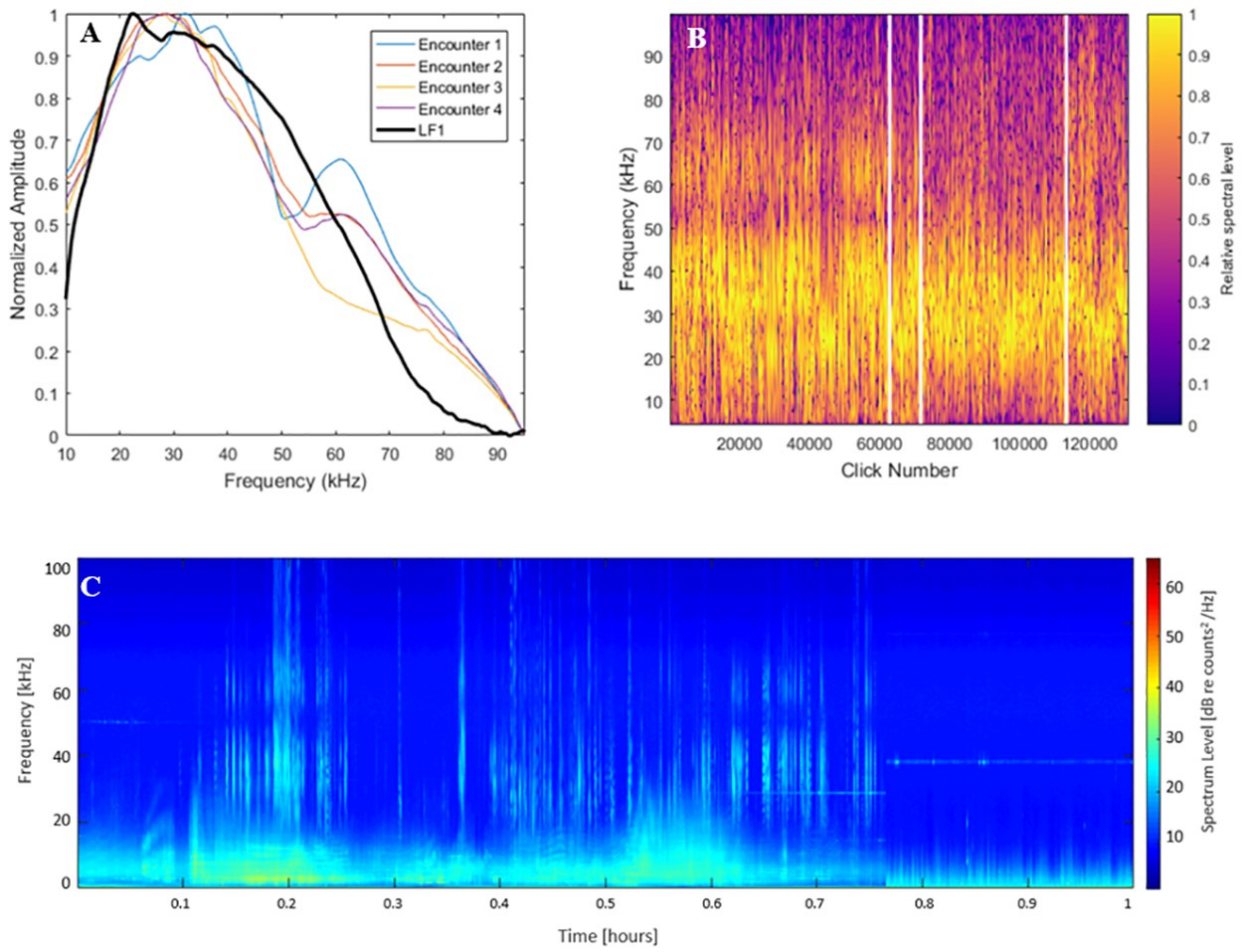
Towed-array *S*. *bredanensis* encounters. Figure depicting (a) mean spectra, (b) concatenated click spectra, and (c) an example long-term spectral average of a towed-array acoustic encounter of verified rough-toothed dolphins. Panel (a) includes the mean type spectra of the LF1 click type for comparison. Delineations in panel (b) (white lines) separate clicks coming from encounters 1–4. Panel c shows a long-term spectral average of raw data from an example encounter coming from the towed array dataset.

**Fig 4:**
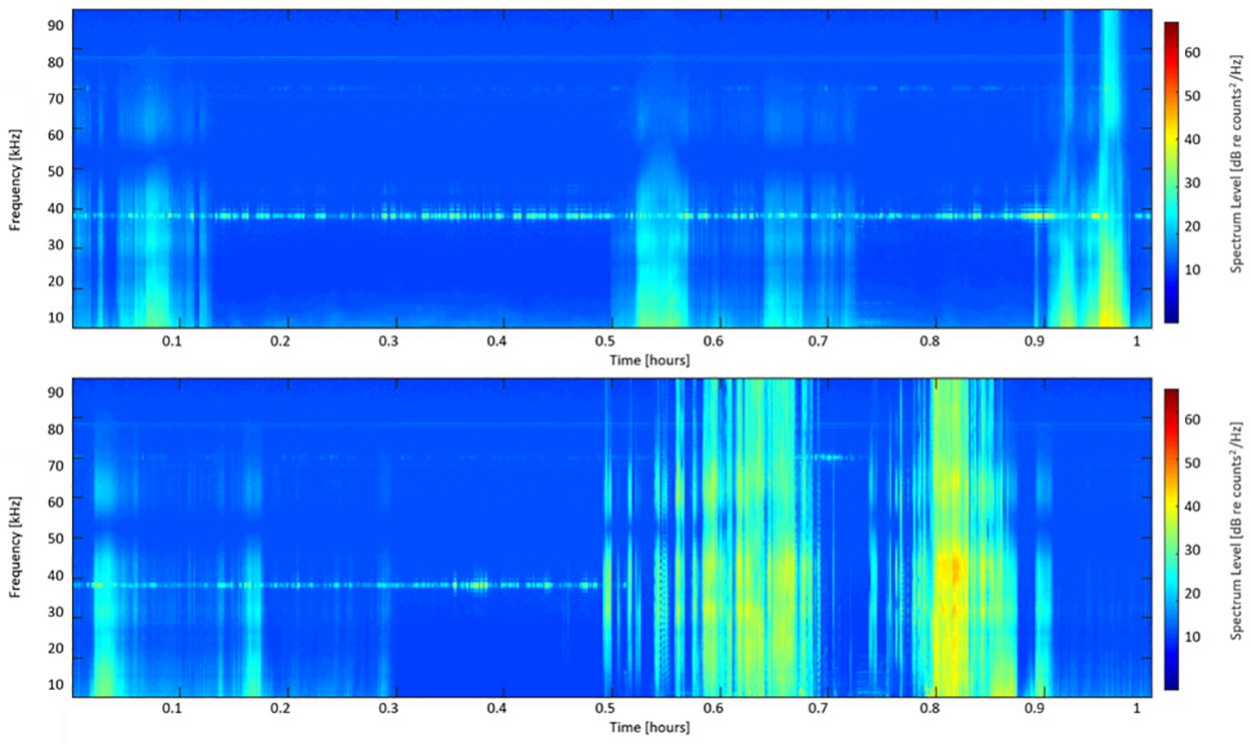
Additional towed-array examples. Long-term spectral average of two different hours of towed-array data, displaying the persistence of a notch in sensitivity at ~ 50 kHz regardless of species present. Panel (a) displays sound data from anthropogenic sources, while (b) displays both anthropogenic noise and a delphinid encounter (starting at ~ 0.5 hours).

Labelled towed array data from HICEAS 2017 supported the hypothesis that LF1 was a rough-toothed dolphin click type. Four acoustic encounters with visually-verified rough-toothed dolphins were compared to LF1 clicks to determine the suitability of this classification. The mean spectra from these encounters were compared to the type spectrum for LF1, and found to be a fairly consistent match across all encounters, though the mean spectra for encounter 1 had a higher-frequency peak and less content in the band from 20–30 kHz when compared to the type example and other encounters (Fig 3). It is notable that a spectral ‘notch’ existed in the towed array data at about 50 kHz. This notch was determined to be an artifact of the data and not related to the clicks presented here due to its persistence across the dataset (Fig 4).

**Fig 5:**
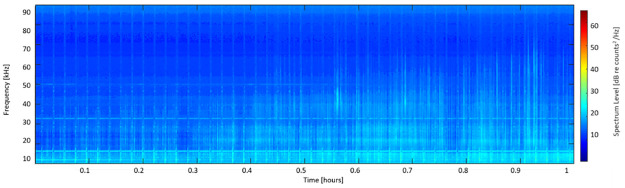
Long-term spectral average of an LF1 encounter. Long-term spectral average of data from 11/13/2015 including clicks labelled as type LF1. A sighting of rough-toothed dolphins occurred about 1 hour after this encounter, approximately 5 km from the location of the HARP.

Additionally, a total of four sightings of rough-toothed dolphin were deemed potentially usable for ground-truthing type LF1 based on proximity to the Kona or Kauaʻi HARPs during times at which the HARPs were recording (distance <10 km from the HARP). Of these, two sightings approximately 5 km from the Kauaʻi site had concurrent LF1 clicks recorded on the HARP (example long-term spectral average, Fig 5); the other two sightings did not have concurrent clicks of any type. For the sighting shown, clicks of the LF1 type began approximately 2.5 hours before the sighting, continuing until about one hour before the sighting. In this example, the increase in energy < 20 kHz towards the end of the window is due to a ship passage. Based on the evidence compiled through these methods, it was concluded that LF1 represented the clicks of the rough-toothed dolphin.

#### C—Short-finned pilot whale

Two click types in the data were likely attributable to the short-finned pilot whale. The first of these types was characterized by two spectral peaks, one at 13.0 kHz, and a more dominant peak at 28.0 kHz. For these peaks, -3 dB bandwidths were 1.5 and 5.0 kHz respectively. The peak ICI for this type was 184 ms (Fig 2C1, Table 1). The second type was characterized by three spectral peaks: two more minor peaks at 13.0 and 18.5 kHz, and one higher amplitude peak at 48.5 kHz. In this case, -3 dB bandwidths were 1.5, 3.0, and 3.0 kHz, respectively. This type had a peak ICI of 206 ms (Fig 2C2, Table 1). An attempt was made to group these types together under one ‘short-finned pilot whale’ class for neural network training and testing purposes; however, classifier performance was improved by leaving the two types as separate classes. Validation for this type description was provided by previous acoustic records and spatial distribution data (Table 2, S1 File).

#### D—Bottlenose dolphin and melon-headed whale

The combined bottlenose dolphin and melon-headed whale (Tt/Pe) echolocation click type was characterized by a dominant peak at 32.5 kHz with a -3 dB bandwidth of 5.5 kHz and an often-present lower frequency peak at 12.5 kHz with a -3 dB bandwidth of 1.5 kHz. Peak ICI for this type was 109 ms (Fig 2D, Table 1). It is worth noting that the lower frequency peak was likely the result of residual energy from whistles that often accompany echolocation clicks of this type. Validation for this mixed type was provided by previous acoustic records, spatial distribution data, and temporal behavior records (Table 2, S1 File).

#### E—Blainville’s beaked whale

The Blainville’s beaked whale echolocation click type was characterized by a dominant higher-frequency peak at 36.0 kHz with a -3 dB bandwidth of 9.0 kHz and a minor, not always present, lower-frequency peak at 24.0 kHz with a -3 dB bandwidth of 2.5 kHz. This click type had a peak ICI of 319 ms (Fig 2E, Table 1). The type was determined to be Blainville’s beaked whale based on previous acoustic records and spatial distribution data (Table 2, S1 File).

#### F—Cuvier’s beaked whale

The Cuvier’s beaked whale echolocation click type was characterized by a dominant peak at 40.0 kHz and lower-amplitude spectral peaks at 17.0 and 24.0 kHz (Fig 2F, Table 1). This click type had a peak ICI of 433 ms. This type distinction was validated using previous acoustic and spatial distribution records (Table 2, S1 File).

#### G—Stenellids

The stenellid echolocation click type was defined by spectral peaks at 18.5 and 50 kHz, with a peak ICI of 48.5 ms (Fig 2G1, Table 1). The lower frequency spectral peak in this type was likely the result of residual energy from underlying whistles. A second type of stenellid clicks was identified in this dataset (Fig 2G2, Table 1), defined by spectral peaks at 25.0 and 39.5 kHz, with a peak ICI of 53.5 ms. However, it was determined that the differences between these two subtypes that lead to their separation during clustering was most likely due to differences in recording equipment. The second type was seen almost exclusively in deployments where the crossover frequency between the low- and high-frequency hydrophones was 25 kHz, which may introduce artificial notches in click spectra, likely causing the peak at 25 kHz seen for this type (Fig 2G2). It would be reasonable to group these types into one stenellid group; however, as with short-finned pilot whale, the two types were left separate due to improved classifier performance. Validation of these types as stenellid was provided based on previous acoustic and spatial distribution data (Table 2, S1 File).

#### H—*Kogia* spp

The *Kogia* spp. click type was defined by a single high-frequency peak at 93.5 kHz and a peak ICI of 90.3 ms (Fig 2H, Table 1). The full spectral shape of these clicks was not captured here as it is above the limit of the bandpass filter used in the original click detection step (100 kHz), but the partial peak captured was indicative of aliasing from higher-frequency (i.e., 125 kHz) *Kogia* spp clicks [54]. Acoustic differentiation between species of *Kogia* is not possible with the available data; hence description of this type must be left at the genus level. Validation for this type was provided by previous acoustic and spatial distribution records (Table 2, S1 File). Information provided in said records suggested that this type was mostly composed of dwarf sperm whale clicks (S1 File).

### Classifier performance

The best performing neural-network based classifier consisted of the following: an input layer, four 512-node fully-connected layers with 50% dropout between each, and a softmax output layer. Networks of this type were trained on a variety of feature combinations, with the best performance (highest accuracy and recall values across classes and sites) resulting from training on clustered 5-minute bin values of peak ICI, mean spectral shape, and mean waveform envelope. Accuracy for this classifier on novel data, which was manually labelled at the 5-minute bin level for network evaluation purposes, was high across classes and sites (> 96% in all cases), with lowest accuracy occurring for false killer whales at the Kona and PHR sites (96.2% and 96.6%, respectively), and rough-toothed dolphin (97.3%) at the Kauaʻi site. The false killer whale class also had the lowest accuracy score for the combined-sites results (96.9%) followed by rough-toothed dolphin (98%). Accuracy was highest for the Cuvier’s beaked whale and *Kogia* spp. classes at the Kona site (99.7%), Blainville’s and Cuvier’s beaked whales at Kauaʻi (99.8%), and Cuvier’s beaked whale at PHR (99.7%); though in the full-site data accuracy was highest for Cuvier’s beaked whale and stenellid type 2 (99.7%) (Table 3).

**Table 3:** Neural network results.

Neural Network Class	Kona				Kauaʻi				PHR				All Sites			
	Accuracy (%)	Recall (%)	Precision (%)	nBins	Accuracy (%)	Recall (%)	Precision (%)	nBins	Accuracy (%)	Recall (%)	Precision (%)	nBins	Accuracy (%)	Recall (%)	Precision (%)	nBins, nClicks
False killer whale	96.2	88.0	**48.5**	324	98.1	93.4	75.9	259	96.6	96.5	**34.9**	86	96.9	91.2	**53.3**	669, 7938
Rough-toothed dolphin	98.4	83.1	83.8	468	97.3	96.7	98.9	4602	98.1	94.1	94.7	801	98	95.3	97.1	5871, 7293
Short-finned pilot whale 1	98.1	89.6	89.0	988	97.8	92.2	79.1	230	98.4	80.6	**58.6**	72	98.1	89.5	84.9	1290, 13271
Short-finned pilot whale 2	98.2	96.3	81.1	463	98.7	66.7	37.5	36	99.2	72.7	25.8	11	98.5	93.7	**74.1**	510, 95262
Tt/Pe	98.5	75.6	**74.9**	213	98.4	94.8	80.5	192	98.2	82.7	91.0	307	98.4	83.8	82.9	712, 3291
Blainville’s beaked whale	99.4	96.0	90.8	227	99.8	98.9	98.9	91	99.5	98.9	99.8	2008	99.5	98.6	98.9	2326, 11713
Cuvier’s beaked whale	99.7	83.3	45.5	6	99.8	N/A	0	0*	99.7	97.3	100	546	99.7	97.1	98.5	552, 271561
Stenellid sp. 1	98.4	97.8	97.9	5840	98.5	98.4	95.6	1219	98.4	94.5	90.3	364	98.4	97.8	97.1	7423, 9802
Stenellid sp. 2	99.5	99.7	93.9	729	99.6	97.8	94.7	183	100	100	96.0	24	99.7	99.4	94.1	936, 824
*Kogia* spp.	99.7	99.6	93.6	250	99.6	100	91.2	52	99.4	100	52.0	13	99.6	99.7	90.2	315, 81444

Results describing the accuracy, recall, and precision on novel, manually-labelled data for each class at each site, as well as for all sites combined. Values less than 75% with >50 bins have been bolded. NBins gives the number of manually labelled positive bins for each type. For full-site results, NClicks gives the average number of clicks in each NBin.

*Manual evaluation of all Kauaʻi data found no clicks of Cuvier’s beaked whale, so recall and precision could not be evaluated.

While accuracy was markedly high for all types, recall and precision presented a more nuanced picture. Lower recall values (i.e. below 75%) indicating that bins of a given type were missed by the network were found for short-finned pilot whale class 2 at Kauaʻi and PHR (Table 3). However, this type is also fairly uncommon at these sites as indicated by a low number of presence bins within the manually labelled dataset for that type (36 bins at Kauaʻi and only 11 bins at PHR, Table 3). Lower precision values, indicating a high presence of false positive bins for a type, were concerning for the false killer whale type at all sites except Kauaʻi, short-finned pilot whale type 2 in the full data, short-finned pilot whale type 1 at PHR, and the bottlenose dolphin/melon headed whale type at Kona (Table 3). Cases where low precision values were likely related to a small number of bins (corresponding to those with low recall as well as Cuvier’s beaked whale at Kona, *Kogia* spp. at PHR, and short-finned pilot whale type 2 at Kauaʻi and PHR) were not considered of concern at this time. The lower precision value for short-finned pilot whale type 2 in the full dataset is likely driven by lower recall at Kauaʻi and PHR, and hence was also not considered concerning.

## Discussion

This study demonstrated the efficacy of machine learning for processing and classifying available large acoustic datasets for odontocete species, in this case in the tropical Pacific islands. Using machine learning methods, it was possible to discriminate the echolocation clicks of five species of odontocetes (false killer whale, short-finned pilot whale, rough-toothed dolphin, Cuvier’s beaked whale, and Blainville’s beaked whale) as well as three additional groups (stenellid dolphins, *Kogia* spp., and bottlenose dolphin/ melon-headed whale). The classification of the LF1 click type as rough-toothed dolphin highlights a unique advantage of the clustering methodology that allows for quantitative grouping of unknown types more easily than manual labelling. While manual identification might allow one to classify data as a known type, or a general ‘unknown’ type, clustering provides a more standardized, facilitated way to determine one or more unknown types in a large dataset, particularly when differences appear to be small yet consistent. As seen with rough-toothed dolphin in this case, combination of click features from an unknown type with other acoustic and sighting records from the region can lead to new classifications and provide insights for species with few previous acoustic descriptions.

It is worth noting, however, that comparisons of acoustic data from multiple recordings requires consideration of system differences. Exact spectral matches between encounters recorded on HARPs versus those recorded by towed acoustic arrays are unlikely due to the differences in both equipment and recording schemes (e.g., recording at the ocean floor versus near the surface, increased noise due to active towing, differences in animal behavior and/or orientation to the receiver). As such, in this study, the general spectral shape of mean spectra from towed array encounters versus the type spectrum from HARP data were given more weight than exact peak values. This study also demonstrates the usefulness of comparing observed relative species presence to sighting records to bolster classifications. While this process did not lead to distinction of the bottlenose dolphin/ melon-headed whale, *Kogia* spp., or stenellid types, the comparisons provide context for what the makeup of these types might be. Similar methodology has recently been applied to delphinid species in the Atlantic Ocean [34].

One potential downfall of this clustering method is the loss of rarer types. By clustering clicks or bins together and setting various pruning thresholds, some connections and clusters were removed from the data, or grouped into larger, more dominant types. In the Hawaiian region, there are at least 18 species of odontocetes [4], but only ten distinct click types were identified using our clustering methods, though sperm whales were purposefully excluded from analysis. Other, rarer species including Risso’s dolphin, *Grampus griseus*, Longman’s beaked whale, *Indopacetus pacificus*, Fraser’s dolphin, *Lagenodelphis hosei*, and pygmy killer whale, *Feresa attenuata*, were not identified in this dataset using these methods. While it is possible that none of these species were present in the dataset, it is more likely that some (primarily pygmy killer whales, given their spatial use off Kona [55]) were present in the record in small numbers and hence not represented by their own cluster. In the case of this study, this lack of representation in the final types resulted in no corresponding class in the neural-net classifier. Detections of these species have hence been unavoidably mislabeled as either a different odontocete class or noise; the effect of this is likely small but difficult to quantify without labelled data for these species.

Noise floor differences between earlier and later models of the HARP recording systems used in this study resulted in differences in detectability for echolocation clicks below 125 dB_pp_ re 1 μPa. Over the course of this study the received level threshold was therefore increased from 115 to 125 dB_pp_ re 1 μPa to mitigate detectability-related artifacts over the 12 year period. This reduced the dataset size by approximately 10%.

The success of the machine learning tools applied here on other datasets may be somewhat dependent on the noise floor. The HARP data used in this case had a very low broad band noise floor (i.e., high signal to noise ratio) for most deployments, facilitating the use of lower amplitude detections for classification. Higher noise recordings, such as those collected using moving towed arrays, may further limit similar analyses to detections with higher received levels.

The development of the input data for the Cuvier’s beaked whale neural network class in this study used examples from Hawaiian HARP data, augmented with additional examples from Southern California. This process seemed to have successfully complemented available Hawaiian examples without causing classifier confusion, as Cuvier’s beaked whale had some of the highest accuracies across all three sites, as well as for all sites combined (> 99% in all cases) (Table 3). Future studies employing these methods might consider the efficacy of augmenting regionally developed classes with additional data from other locations, particularly in the case of species that are not represented in local clusters but are known to be present. When augmenting existing regional classes with additional global examples, researchers must also be wary of species whose echolocation clicks have shown significant regional variation [56], as well as species that produce multiple, distinct types of echolocation clicks [57], which should perhaps not be combined together for classification purposes.

For other classes, training and testing data were augmented using noise to reach a total of 5000 example bins. It is possible that this may introduce artifacts into the data, which can then be learned by the network [35]. However, this process seemingly did not cause issues for the classifier created in this study; inspection of augmented data revealed no noticeable spectral artifacts, and classification accuracy was high amongst both augmented and non-augmented types. As an example, one can compare performance of the non-augmented stenellid type 1 class to performance of the augmented Blainville’s beaked whale class on novel data (Table 3). In all three cases, accuracy and recall across sites was >94%, suggesting that augmenting input features did not have harmful effects on classifier performance. The classifier produced in this process demonstrated a high degree of classification accuracy on novel data across sites and classes, and fairly high (> 66% in all cases) values for recall (Table 3). The success of these augmentation techniques imply that these methods can be useable on smaller datasets. However, network training on less than a few hundred examples of determined types, with no ability to augment these types using additional data, should proceed with caution. The clustering and neural network steps employed here were developed to expedite processing of large acoustic datasets and are best suited to this task.

In some cases, examination of confusion matrices provides useful context for performance metric values. For false killer whales, confusion matrices at all sites revealed that the low precision seen for this type was mainly due to bins of noise being mislabeled as false killer whale (Tables 3–6). This result was most likely due to the spectral similarities between the false killer whale click type and detections attributed to boats and sperm whales that were part of the input noise class, particularly after applying a 10 kHz high-pass filter. This issue was less pronounced at Kauaʻi, where false killer whales were more common relative to noise detections (Table 3; Table 6).

**Table 4:** Classifier confusion Matrix- Kona.

	False killer whale	Rough-toothed dolphin	Short-finned pilot whale 1	Short-finned pilot whale 2	Tt/Pe	Blainville’s beaked whale	Cuvier’s beaked whale	Stenellid 1	Stenellid 2	*Kogia* spp.	Noise
False killer whale	285	0	25	8	1	0	0	0	0	0	5
Rough-toothed dolphin	1	389	19	14	2	1	0	33	3	0	6
Short-finned pilot whale 1	45	23	885	5	6	2	0	14	2	0	6
Short-finned pilot whale 2	6	3	5	446	0	0	0	2	0	0	1
Tt/Pe	0	14	16	11	161	1	0	3	7	0	0
Blainville’s beaked whale	0	0	0	1	1	218	1	3	0	1	2
Cuvier’s beaked whale	0	0	0	0	0	0	5	0	0	0	1
Stenellid 1	1	24	19	17	35	3	5	5713	16	1	6
Stenellid 2	0	1	0	1	0	0	0	0	727	0	0
*Kogia* spp.	0	0	0	0	1	0	0	0	0	249	0
Noise	250	10	25	47	8	15	0	67	19	15	3662

Confusion matrix showing the number of 5 minute bins (from novel data not used in training/testing/validation) labelled as each class. Diagonal cells across classes show the number of correctly labelled positive bins for that class. Cell(i,j) is the number of bins of i that were labelled j by the network.

**Table 5:** Classifier confusion Matrix- Pearl and Hermes Reef.

	False killer whale	Rough-toothed dolphin	Short-finned pilot whale 1	Short-finned pilot whale 2	Tt/Pe	Blainville’s beaked whale	Cuvier’s beaked whale	Stenellid 1	Stenellid 2	*Kogia* spp.	Noise
False killer whale	83	1	2	0	0	0	0	0	0	0	0
Rough-toothed dolphin	11	754	21	1	4	0	0	7	0	2	1
Short-finned pilot whale 1	11	0	58	0	2	0	0	1	0	0	0
Short-finned pilot whale 2	1	1	0	8	1	0	0	0	0	0	0
Tt/Pe	0	33	0	0	254	0	0	20	0	0	0
Blainville’s beaked whale	0	0	8	7	2	1986	0	1	0	2	2
Cuvier’s beaked whale	0	0	4	7	1	1	531	0	1	0	1
Stenellid 1	0	4	2	0	14	0	0	344	0	0	0
Stenellid 2	0	0	0	0	0	0	0	0	24	0	0
*Kogia* spp.	0	0	0	0	0	0	0	0	0	13	0
Noise	132	3	4	8	1	3	0	8	0	8	1187

Confusion matrix showing the number of 5 minute bins (from novel PHR data not used in training/testing/validation) labelled as each class. Diagonal cells across classes show the number of correctly labelled positive bins for that class. Cell(i,j) is the number of bins of i that were labelled j by the network.

**Table 6:** Classifier confusion Matrix- Kauaʻi.

	False killer whale	Rough-toothed dolphin	Short-finned pilot whale 1	Short-finned pilot whale 2	Tt/Pe	Blainville’s beaked whale	Cuvier’s beaked whale	Stenellid 1	Stenellid 2	*Kogia* spp.	Noise
False killer whale	242	14	1	0	0	0	0	0	0	0	2
Rough-toothed dolphin	20	4450	38	7	29	0	1	44	9	0	4
Short-finned pilot whale 1	5	9	212	0	4	0	0	0	0	0	0
Short-finned pilot whale 2	2	7	1	24	0	0	0	0	1	0	1
Tt/Pe	0	3	5	0	182	0	0	2	0	0	0
Blainville’s beaked whale	0	0	0	1	0	90	0	0	0	0	0
Cuvier’s beaked whale	0	0	0	0	0	0	0	0	0	0	0
Stenellid 1	0	4	1	3	8	0	0	1199	0	0	4
Stenellid 2	0	1	0	1	1	0	1	0	179	0	0
*Kogia* spp.	0	0	0	0	0	0	0	0	0	52	0
Noise	50	12	10	28	2	1	0	9	0	5	1088

Confusion matrix showing the number of 5 minute bins (from novel Kauaʻi data not used in training/testing/validation) labelled as each class. Diagonal cells across classes show the number of correctly labelled positive bins for that class. Cell(i,j) is the number of bins of i that were labelled j by the network.

Other recent classification efforts of false killer whale have found success using a variety of vocalizations instead of only echolocation clicks, however, accuracy was slightly reduced when attempting to classify multiple species instead of only false killer whales and a conglomerate outgroup of other odontocetes [26]. Due to the regional significance of this species, and their rarity in the dataset, manual data verification was required to remove false positives from false killer whale time series before discussion of relative acoustic presence in this dataset, as mentioned in the results section of this study. This adjusted methodology highlights how the classifier can still be used to efficiently select time periods of presence for this type (i.e., high recall). Though the false positive rate on these detections was high (i.e., low precision), the additional manual checking required to remove these was much faster than manual logging of the entire dataset.

Low precision in classes other than false killer whales and types with few bins evaluated (e.g., Cuvier’s beaked whale at Kona; Table 3) occurred primarily for short-finned pilot whale and bottlenose dolphin/melon headed whale types (Table 3). Though the number of manually labelled bins for short-finned pilot whale class 1 at PHR exceeded the threshold for evaluation (50 bins), it was still fairly low (nBin = 72; Table 3). Based on precision values for this type at the other two sites, low bin number is considered the most likely reason for lower precision observed in this case. For the bottlenose dolphin/melon-headed whale type, confusion exists primarily with short-finned pilot whale, stenellid, and rough-toothed dolphin types (Tables 4–6). This is potentially due to several factors. Differences between the structure of the training set, which contained equal proportions of each type, and the real data, where types may be less common at certain sites than the network expects (e.g., rough-toothed dolphin at Kona) may increase confusion. Additionally, the ICI distribution and overall frequency range of rough-toothed dolphin, short-finned pilot whale, and bottlenose dolphin/ melon-headed whale clicks were fairly similar, as was the spectral content of rough-toothed dolphin, bottlenose dolphin/ melon-headed whale, and stenellid clicks, particularly in low received level encounters where the higher frequency content of stenellid clicks was not as prevalent. Confusion amongst these classes was lower at Kauaʻi than at Kona, potentially due to the lower level of vessel presence at this site. The Kona site has an overall much higher noise background than the Kauaʻi site related to the commonality of ships and echosounders. These noise sources may alter spectral content of click mean spectra as well as ICI distributions by introducing false positive detections, hence increasing network confusion. There is not much that can be done with the current classifier structure to reduce this confusion, though it is notable that even in the most drastic case (i.e., bottlenose dolphin/melon headed whale at Kona), the precision value was still fairly high (74.9%). Network confidence, which accompanies all labels from the neural network used in this study, could potentially be used in future applications of this dataset to improve upon these false positive rates by only using detections above a certain confidence threshold.

Using the machine learning methods applied in this study, we were able to develop a catalogue of click types for the Hawaiian Islands region and attribute those click types to species, including the novel description of a click type for rough-toothed dolphins. We were then able to develop and implement a neural-net based classifier, from which we were able to label encounters with 8 or more species of odontocetes in 15 instrument years of passive acoustic data. The success of this classifier in labeling passive acoustic data from multiple sites demonstrates its efficacy for analyzing existing and future acoustic datasets from this region, as well as potentially from other regions where these species are thought to be present. Future work related to improving the success of these methods in identifying and classifying echolocation clicks might consider a tiered approach, in which original clustering and labelling take place at a more generalized level (e.g., ‘unidentified dolphin’, ‘beaked whale’, ‘ship’). Then, parsing of subtypes could be completed using additional clustering as well as other methods that have proved useful here such as comparison to known click records, species patterns, and auxiliary data. Such a method would likely minimize misclassifications as well as avoid the issue of species without a specific class being unavoidably mislabeled as a different odontocete or potentially as noise. At present, however, a usable workflow has not yet been developed for the method proposed above.

In this paper, knowledge of rough-toothed dolphin diel behavior was used as additional evidence in the attribution of click type LF1 to this species. Further research using the timeseries produced in this study may find that examining diel patterns helps bolster the classifications made here. This may be particularly true for the stenellid type, as spinner dolphins are active nearly exclusively at night and spend their days in shallow resting bays [58], whereas spotted dolphins, while still more active at night, are somewhat active during the day as well [3, 59]. In addition to this, Blainville’s beaked whales have demonstrated diel and lunar variation in activity [20, 60, 61], short-finned pilot whales have been noted to move inshore/offshore in relation to the lunar cycle [62], and differences in diel presence among sites may help determine the makeup of the bottlenose dolphin/melon headed whale class. For *Kogia* spp. and some others, comparisons to timeseries produced using subsets of this HARP dataset (e.g., [63]) will provide additional useful context for those derived using the methods of this paper. For false killer whales, marked presence during night-time hours may help explain the mismatch between rare sightings and common presence in the Kauaʻi HARP dataset (31% of days with presence); this could also be investigated using existing satellite tag data [64, 65] to see whether they are more likely to use the area off western Kauaʻi during night-time hours. Work on describing and exploring some of these comparisons is ongoing and will be addressed more completely in a future paper. The records developed here can also be used in species monitoring efforts as well as to answer complex questions about animal behavior, habitat requirements, and ecosystem relationships of odontocetes in the Hawaiian Islands.

## Supporting information

S1 Table Recording schedule. Recording schedule for deployments from all sites. For one deployment (denoted by an asterisk in the duty cycle column), the duty cycle was inconsistent due to a system malfunction, resulting in about 1/3 time on. Depth is given as the seafloor depth at the deployment location, to the nearest 10 meters. Deployments with a 25 kHz crossover between the low and high frequency hydrophones are bolded.
(PDF)

S1 File Supporting information for click type validation. Information from relevant previous studies used to validate the attribution of click types in this study to known species or species groups.
(DOCX)
