# Supplementary material for: Discriminating and classifying odontocete echolocation clicks in the Hawaiian Islands using machine learning methods
Source: PLoS One. 2022 Apr 12;17(4):e0266424. doi: 10.1371/journal.pone.0266424 (PMC9004765; doi:10.1371/journal.pone.0266424)
Supplement: S1 Table — Recording schedule for deployments from all sites. For one deployment (denoted by an asterisk in the duty cycle column), the duty cycle was inconsistent due to a system malfunction, resulting in about 1/3 time on. Depth is given as the seafloor depth at the deployment location, to the nearest 10 meters. Deployments with a 25 kHz crossover between the low and high frequency hydrophones are bolded. (PDF) [file pone.0266424.s001.pdf]

Deployment	Sampling Frequency (kHz)	Duty Cycle (minutes on/total minutes)	Latitude, Longitude (decimal degrees)	Instrument Depth (nearest 10 meters)	Start Date	End Date
Kona01	200	N/A	19.582, -156.015	730	2007-08-11	2007-10-04
Kona02	200	5/8	19.581, -156.015	730	2008-04-19	2008-07-04
Kona03	200	5/15	19.582 -156.015	730	2008-07-08	2008-10-15
Kona05	200	5/5	19.578, -156.014	550	2009-02-10	2009-04-01
Kona06	200	5/15	19.583, -156.016	750	2009-04-23	2009-08-18
Kona07	200	N/A	19.582, -156.015	720	2009-10-25	2009-12-15
Kona08	200	5/12	19.581, -156.015	750	2009-12-20	2010-03-05
Kona09	200	N/A	19.581, -156.016	750	2010-05-01	2010-06-16
Kona10	200	5/8	19.582, -156.015	720	2010-09-30	2011-03-12
Kona11	200	5/8	19.582, -156.015	720	2011-05-12	2011-10-22
Kona13	200	5/10	19.583, -156.015	720	2012-05-25	2012-07-18
Kona14	200	5/10	19.583, -156.016	720	2012-11-17	2013-02-28
Kona15	200	5/15	19.583, -156.016	720	2013-05-25	2013-10-19
**Kona16**	200	5/15	19.583, -156.016	720	2013-10-23	2014-04-03
**Kona17**	200	N/A	19.583, -156.016	720	2014-03-25	2014-07-14
**Kona18**	320	N/A	19.583, -156.016	720	2014-07-28	2014-10-12
**Kona19**	320	N/A	19.583, -156.016	720	2014-12-06	2015-03-06
**Kona20**	320	N/A	19.583, -156.016	720	2015-04-25	2015-08-18
**Kona22**	200	N/A	19.583, -156.016	720	2015-11-07	2016-03-19

**Kona23**	200	N/A	19.583, -156.016	720	2016-07-04	2016-09-14
**Kona25**	320	5/12*	19.583, -156.016	740	2017-05-01	2017-07-10
**Kona26**	200	N/A	19.582, -156.015	740	2017-07-12	2017-10-25
**Kona27**	200	N/A	19.583, -156.016	750	2017-10-26	2018-04-25
**Kona28**	200	N/A	19.583, -156.016	730	2018-04-29	2018-11-19
**Kona29**	200	N/A	19.583, -156.016	740	2018-11-23	2019-03-31
Kona30	200	N/A	19.583, -156.015	700	2019-04-04	2019-09-29
Kauai01	200	5/20	21.953, -159.887	720	2009-10-08	2010-05-13
Kauai02	200	N/A	21.954, -159.890	730	2010-06-04	2010-08-20
**Kauai05**	200	5/7	21.949, -159.888	730	2016-07-09	2017-08-09
PHR01	200	5/20	27.725, -175.638	770	2009-10-20	2010-05-24
PHR02	200	N/A	27.727, -175.632	750	2010-06-01	2010-09-17
PHR04	200	N/A	27.725, -175.638	770	2011-04-12	2011-07-29
PHR05	200	5/8	27.725, -175.638	770	2011-08-15	2012-01-07
**PHR08**	200	5/20	27.742, -175.559	970	2014-09-12	2015-07-16
PHR09	200	5/30	27.742, -175.560	960	2015-10-15	2016-08-14
PHR10	200	5/30	27.741, -175.560	960	2016-08-20	2017-03-14
PHR11	200	5/30	27.727, -175.555	1070	2017-08-20	2018-03-05
PHR12	200	5/30	27.730, -175.554	1150	2018-10-15	2019-06-10
