# Supplementary material for: Discriminating and classifying odontocete echolocation clicks in the Hawaiian Islands using machine learning methods
Source: PLoS One. 2022 Apr 12;17(4):e0266424. doi: 10.1371/journal.pone.0266424 (PMC9004765; doi:10.1371/journal.pone.0266424)
Supplement: S1 File — Information from relevant previous studies used to validate the attribution of click types in this study to known species or species groups. (DOCX) [file pone.0266424.s002.docx]

**A — False killer whale validation**

The assignment of the false killer whale click type was based on previous descriptions of echolocation clicks of this species [26]. Relative acoustic presence of this type was highest at Kauaʻi (31% of days had presence), followed by Kona (5%), and then PHR (3%). Despite the presence of three endemic stocks of this species in the region [27], sightings of false killer whales have been fairly rare; a comprehensive study of the Main Hawaiians Islands based on small boat surveys from 2000-2012 found that false killer whales made up a total of only 0.4% of sightings near Kauaʻi, and 2.2% of sightings leeward off Hawaiʻi Island (i.e., near the Kona HARP)[4]. Habitat-based density models of false killer whales suggest a density of 0.0005-0.001 animals/km^2^ in the vicinity of all three HARPs [28]. Satellite tagging of the main Hawaiian Islands insular stock of false killer whales has also suggested that neither the Kona nor Kauaʻi HARP locations are high-use areas for the species [29].  Further investigation into these results is needed to illuminate why relative acoustic presence of this type is higher than would be predicted based on previous false killer whale research.

**C — Short-finned pilot whale validation**

The main peak (28.0 kHz) of the first short-finned pilot whale click type fell within the range of previously described short-finned pilot whale clicks from the region but did not have typical banding at 18 kHz [26]. This type was more similar to the presumed pilot whale click type determined by Frasier et al. (2017) [16]. The second of these types had lower peaks (13.0 and 18.5 kHz) corresponding to the spectral banding often associated with short-finned pilot whale echolocation clicks, and a generally comparable overall spectral shape (Fig. 2C2), although its third peak (48.5 kHz) was higher in frequency than the high-frequency peak described by Baumann-Pickering et al. (2015) [26]. These types often co-occurred in the data, with detections of type 1 generally having a lower received level and occurring near the start or end of an encounter, enveloping detections of type 2. Combined labels of the two types matched well with encounters ascribed to short-finned pilot whales in Baumann-Pickering et al. (2015). The two types also had similar relative acoustic presences at Kona (~60% of days in both cases), where both were more common compared to their presence at other sites. The two types were less common at Kauaʻi (27% and 52%) and PHR (18% and 25%).

The trend in these acoustic presences matched well with the sighting record, in which sightings of short-finned pilot whales were more common leeward of Hawaiʻi Island than at other main Hawaiian Island locations (~30% vs <15% in all other cases,[4]). The Kona and Kauaʻi HARPs are within the modelled area of highest estimated density for this species (1.6 – 10 animals/ 100 km^2^); the PHR site is in the second-highest density estimate tier (0.8 – 1.6 animals/ 100 km^2^) [37]. Both types may be variations of echolocation clicks produced by the short-finned pilot whale. There is no evidence at present that suggests any relationship between these types and the different insular or pelagic populations, or multiple communities of insular short-finned pilot whales that exist in Hawaiian waters [38], though differing dialects with regards to social calls have been noted in these communities [39].

**D — Bottlenose dolphin and Melon-headed whale validation**

Peak frequencies at 12.5 and 32.5 kHz and a modal ICI value of 109 ms for this type were comparable to previous records of species-specific echolocation clicks of both bottlenose dolphins and melon-headed whales [40]. These clicks occurred with a strong diel pattern, with clicks being more prevalent at night. This may suggest that the type was predominantly melon-headed whale clicks [41-42]. While this suggests that the type represented melon-headed whale, it does not rule out the possibility that bottlenose dolphin echolocation clicks were also included in this type.

Sighting data from the region has suggested that melon-headed whales are more commonly found leeward of Hawaiʻi Island than near the other two sites (3% of sightings leeward of Hawaiʻi vs. 1.3% of sightings near Kauaʻi)[4]. In contrast, bottlenose dolphins represent a fairly large percentage of sightings across the main Hawaiian Islands, particularly near Kauaʻi (~25% of sightings, ~ 6% leeward of Hawaiʻi [4]). Density models of bottlenose dolphin presence suggest that all three HARP sites, in particular the PHR site, have the potential to be areas of high-density for this species [37]. Melon-headed whales’ use of offshore eddies as foraging grounds [43] implies presence at nearshore HARP sites would be low. The relative acoustic presence of this type does not provide strong evidence to differentiate which of the two species is most likely to have produced these clicks; presence is even across the three sites (~35% of days in all cases). Due to this lack of strong evidence for either species, this type was considered to be an unknown combination of the two, though it might also be strictly one or the other.

**E — Blainville’s beaked whale validation**

The spectral peak values (24.0 and 36.0 kHz) found for this type were consistent with previous records of Blainville’s beaked whale echolocation clicks [17]. Relative acoustic presence of this species was highest at PHR (~93 % of days) followed by Kona (~38 %) and Kauaʻi (~ 23%). Previous studies of Blainville’s beaked whales near Hawaiʻi have resulted in few sightings leeward of Hawaiʻi Island and Kauaʻi (2.5%, 2.2% respectively, [4]), though it is notable that sighting percentages for this species as well as other deep, long-duration divers are not very comparable to sighting rates of other species.

Known foraging hotspots for Blainville’s beaked whales near Kona [44-45] potentially provide a reason for the higher relative acoustic presence noted there Kauaʻi. Sightings in the Northwestern Hawaiian Islands have been limited to large-spatial scale visual surveys, for which the effort is not comparable to that which has occurred in the main Hawaiian Islands. However, results of these surveys have included sightings of Blainville’s beaked whales in the general vicinity of PHR [35], and previous acoustic research using HARP data from this site also noted high presence of the species [46].

**F — Cuvier’s beaked whale validation**

The spectral peaks of this click type (17.0, 24.0, and 40.0 kHz) were similar to Baumann-Pickering et al. (2013) [17]. Similar to the Blainville’s beaked whale type, Cuvier's beaked whale presence was highest at PHR (~57% of days), and lower both at Kona and Kauaʻi (12% and 2%, respectively). This general trend was corroborated by sighting records of Cuvier’s beaked whales (0% of sightings around Kauaʻi, ~4% of sightings leeward of Hawaiʻi Island [4]). A Hawaiʻi Island-associated population exists [45], and there have been no documented sightings off Kauaʻi [3].

**G — Stenellid validation**

The dominant peak and ICI of stenellid types (39.5 and 50.0 kHz) were consistent with previous studies of stenellid dolphin echolocation clicks [40, 47]. In the Hawaiian Islands, relevant stenellid species are the pantropical spotted dolphin, spinner dolphin, and the striped dolphin*.* Based on sighting information and NOAA surveys of the islands, it is likely that the stenellid click type represented primarily spinner and pantropical spotted dolphins, as striped dolphins seem to prefer deeper water and represent very few of the sightings from this region, particularly in depths <3000 m (<3% of sightings total) [4, 28, 35]. Relative acoustic presence of this type was high at Kona (95% of days with presence), and lower at Kauaʻi and PHR (79% and 48%, respectively). Higher presence at Kona fits with sighting records of pantropical spotted dolphins, which have been seen an order of magnitude more off Hawaiʻi Island than off Kauaʻi [48]. In contrast, low predicted density of spotted dolphins near PHR [37] and the presence of an island-associated population of spinner dolphins recognized at that atoll [49] may suggest that stenellid clicks from PHR mostly represent spinner dolphins.

**H — *Kogia* spp. validation**

Based on previous records, this type likely represented the genus *Kogia*, including both the dwarf and pygmy sperm whale [9, 50]*.* Relative acoustic presence of this type was highest at Kona (45% of days), and lower at both Kauaʻi and PHR (24% and 20%, respectively). Sightings of *Kogia* spp. are uncommon, as both species are especially cryptic [4]. Sighting rates of dwarf sperm whales are more than three times higher in the lee of Hawaiʻi Island than off Kauaʻi, and sighting rates off Hawaiʻi Island are particularly high in similar depths to the HARPs [51]. Sighting rates of dwarf sperm whales are more than an order of magnitude higher than pygmy sperm whales around the main Hawaiian Islands sightings, and they are seen in significantly greater depths [4]. These findings in conjunction with higher relative acoustic presence at Kona suggest that the *Kogia* spp. type was likely composed of mostly dwarf sperm whale clicks.
